# Human Genetic Diseases Linked to the Absence of NEMO: An Obligatory Somatic Mosaic Disorder in Male

**DOI:** 10.3390/ijms23031179

**Published:** 2022-01-21

**Authors:** Alessandra Pescatore, Ezia Spinosa, Carmela Casale, Maria Brigida Lioi, Matilde Valeria Ursini, Francesca Fusco

**Affiliations:** 1 Institute of Genetics and Biophysics “Adriano Buzzati-Traverso”, IGB-CNR, Via P. Castellino, 111, 80131 Naples, Italy; alessandra.pescatore@igb.cnr.it (A.P.); ezia.spinosa@igb.cnr.it (E.S.); carmela.casale@igb.cnr.it (C.C.); francesca.fusco@igb.cnr.it (F.F.); 2 Department of Science, University of Basilicata, Viale dell’Ateneo Lucano, 85100 Potenza, Italy; maria.lioi@unibas.it

**Keywords:** genetic mosaicism, NF-κB, NEMO/IKKγ, cell death, inflammatory disease

## Abstract

De novo somatic mutations are well documented in diseases such as neoplasia but are rarely reported in rare diseases. Hovewer, severe genetic diseases that are not compatible with embryonic development are caused exclusively by deleterious mutations that could only be found as mosaic and not as inherited mutations. We will review here the paradigmatic case of Incontinentia Pigmenti, a rare X-linked dominant disease caused by deficiency of the NEMO (also called IKKgamma) protein, which plays a pivotal role in tissue homeostasis. The loss-of-function mutations of *NEMO* are embryonically lethal in males while females survive because of unbalanced X-inactivation due to NEMO wild type (WT) expressing cells survival despite of NEMO mutant expressing cells. The few surviving IP males are obligatory mosaic mutants with the typical clinical presentation of IP in female. Indeed, the IP pathogenesis in the female and most likely also in the male somatic mosaics is based on the cellular effects of an impaired NEMO activity, but in the context of the interaction of genetically different cells in the affected tissue, which might underline the inflammatory status.

## 1. Introduction

Disease-causing mutations often originate during embryogenesis after the fertilization and zygote formation. These post-zygotic mutations lead to individuals who are mosaic, with only a subset of their cells harboring the mutation. These mutations are de novo, as they did not exist in the parents of the affected individuals but they are often termed somatic mutations because only somatic cells, most frequently skin or blood, are used to perform genetic analyses [1,2]. Indeed, a suspect of germline mosaicism occur only if a somatic mosaicism is discovered, or if an identical de novo mutation is found in the progeny.

**Figure 1:**
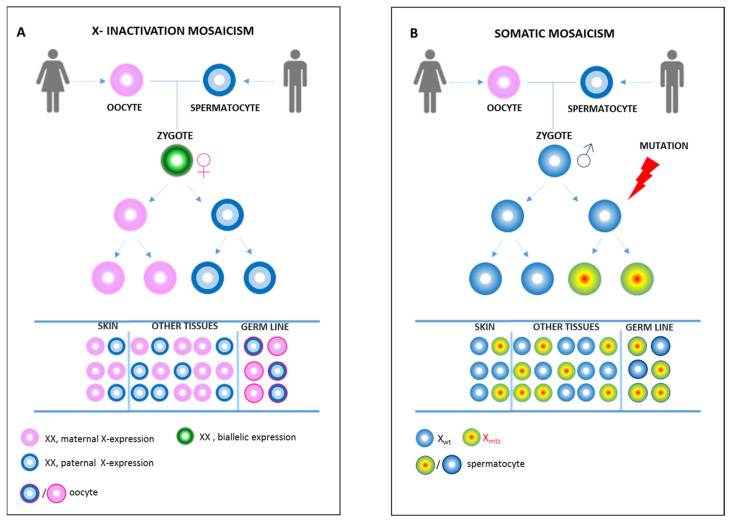
X-inactivation mosaicism or somatic mosaicism. (**A**) X-chromosome inactivation in females is a random event. Once the choice for the inactivation of either the maternal or paternal X-chromosome is made, it is stably inherited by all daughter cells. Therefore, the females are physiological mosaic. The extent of mosaicism in each tissue is made up by selection of cells expressing paternal or maternal X chromosome. This determines a phenotypic condition indistinguishable from the somatic mosaicism in (**B**). An early post-zygotic mutation (X mtz) causes the mosaicism in most or all tissues of the body (including skin, germline, and other tissues), with only a portion of cells in each tissue harboring the mutation.

However, mosaicism in human females is physiologically generated by random X-Chromosome Inactivation (XCI) to balance the X-linked transcriptional dosages between the sexes. When a female is heterozygous for a mutation in a X-linked gene, her tissues are composed of a mosaic of cells either not-expressing/inactivating or expressing/non-inactivating the WT allele (Figure 1A) [3,4].

A paradigmatic example of disease linked to mosaicism is that caused by mutation in the *NEMO* (Nuclear factor κB, Essential MOdulator, also called IKBKG) gene (GenBankNM 003639.3, OMIM300248), due both to its X chromosome localization and to the function of the NEMO protein. NEMO is the essential regulatory subunit of IKK, the kinase complex that induces pro-survival NF-κB activation. Although, several mutations have been reported in genes associated with NF-κB dysfunctions, only mutations in *NEMO* gene have resulted of particular interest due to the complexity of genetic and functional aspects that underline inflammatory processes [5,6].

The mutations of *NEMO* are associated to two clinically different inherited genetic diseases, Anhidrotic Ectodermal Dysplasia, with ImmunoDeficiency (EDA-ID, OMIM#300291) and Incontinentia Pigmenti (IP, OMIM#308300), depending on the genetic state and on their effects on the impairment of NF-κB activation. EDA-ID patients, always males, are hemizygous for a *NEMO* mutation that always preserve a residual NF-κB activation (hypomorphic mutations) [7,8]. Instead, the complete Loss-of-Function (LoF) mutation of *NEMO* is lethal at the embryonic stage of a hemizygous male, while a heterozygous female presenting with IP disease survives because of the XCI [9]. In almost 80% of IP patients, a recurrent deletion of the *NEMO* gene occurs (exon 4–10 deletion), leading to a truncated and faulty NEMO protein. IP is always associated with the establishment of a critical inflammatory response that occurs in the neuroectodermal tissues during development or very early after birth, as evidenced in the skin of a child with IP [10,11,12]. Recent evidences have provided a clear indication that the NEMO deficiency in IP can be associated also to autoimmunity [13].

## 2. What Distinguishes an EDA-ID Male from an IP Male?

In agreement with the well-documented knowledge that complete absence of NEMO is incompatible with life since the early phase of embryonic development, patients with EDA-ID have *NEMO* mutations causing only partial loss of NF-κB activation. EDA-ID presents life-threatening infections combined with ectodermal dysplasia, characterized by rare conical teeth, sparse scalp hair, frontal bossing, and the absence of sweat glands (hypohidrotic). The most severe symptom, always presented in EDA-ID male patients, is an immunodeficiency due to the impaired T and B cell development and function [14,15].

To reinforce the model of incompatibility with life of NEMO absence, some surviving males with the IP phenotype have been reported [16]. Male patients carrying LoF *NEMO* mutation can be classified into two categories that could explain their survival: (1) abnormal karyotypes and (2) mosaicism. The first mechanism has been ascertained only in eight cases of male with Klineferter syndrome with XXY chromosomes and IP phenotype [16,17,18,19,20,21,22,23]; the second mechanism, a somatic mosaicism for the *NEMO* LoF mutation, has been proposed for the majority of IP males as the only possible cause of the disease [24,25]. IP in males, due to post zygotic mutation is similar to IP in female, presenting symptoms affecting the same neurocutaneous tissues and a general post-natal inflammatory condition of the skin. Contrary to EDA-ID, in IP-males immunodeficiency or recurrent bacterial infections have never been reported. Therefore, the model of IP pathogenesis in male and in female is based on the cellular effects of an impaired NEMO activity, but in a context of the interaction of genetically different cells in the affected tissue: cells expressing NEMO and being able to respond to NF-κB stimuli and cells unable to orchestrate that response. 

Finally, while EDA-ID associated *NEMO* mutations are constitutive and detectable in all tissues, the postzygotic *NEMO* mutations in the IP males are generally undetectable in blood and are present only in the skin biopsy obtained from affected inflammatory lesions. Recently, mosaicism for *NEMO* in the germline of two phenotypic IP males have been described, which raises the problem of potential transmission of the IP disease to the offspring [26,27].

## 3. Phenotype Associated to a Mosaic NEMO Deficiency: Differences and Similarities between Mosaic IP Males and IP Females

Female patients with incontinentia pigmenti presented with neurocutaneous syndrome, affecting different tissues at different degree (skin, teeth, hair, nails, eyes, and the central nervous system—CNS). Since the discovery of the disease, the minimal criteria for IP diagnosis have been the typical cutaneous lesions, starting in the neonatal period and naturally evolving in four successive inflammatory stages (vesicular rash, stage 1; verrucous lesions, stage 2; patches of hyperpigmentation skin, stage 3; patches of atrophic hypopigmented skin, stage 4). Clinical phenotype includes: ophthalmologic defects present in 36–30% of IP female (reviewed in a meta-analysis by [28] and reported by [11] in the largest case report, respectively) such as: retinal detachments, retinal vascular anomalies, cataracts and strabismus/vision defect, microphthalmia, optic nerve atrophy; odontologic defects in 54–43% of cases ([29] and [11], respectively) which includes: delayed primary or permanent dentition, cone/peg-shaped teeth, and neurologic defects: seizures, motor and mental retardation, spastic paralysis, hypotonia, hypertonia, cerebral atrophy, hydrocephaly, microcephaly, white matter alterations, cortico/subcortical atrophy, stroke, hemorrhages. Central nervous system (CNS) anomalies are the life-threatening aspects of the disease, reported in about 31% of female patients and leading to major morbidity and even mortality [11,30,31]. In general, immunodeficiency or recurrent infection has only been reported very rarely in women with IP [32].

In general, the IP disease presents with a wide heterogeneous phenotype. The IP females have an unbalanced X-chromosome inactivation mosaicism due to the survival of cells expressing the WT *NEMO* allele, while the cell expressing the mutant *NEMO* allele are prone to death, at least in peripheral blood and skin tissues, which are commonly tested. The mosaicism ranged from 50/50 to 1/99 (NEMO mutant cells/NEMO WT cells) in peripheral blood, depending on the age of the patient. Because of the selective death of NEMO deficient cells, the mutated NEMO protein or the absence of protein is undetectable in the above-mentioned tissues. Accordingly, the identification of the disease-causing missense mutations or mutations causing alterations of the splicing of the NEMO mRNA is not detectable using the cDNA sequencing method [33]. 

The phenotype of IP in males was qualitatively indistinguishable from that in females, in terms of most affected tissues, frequencies, and type of anomalies. In Table 1 we presented data from the literature covering the period from 2013 to 2020 (Table 1 refs. [34,35,36,37,38,39,40,41,42,43,44,45,46,47,48,49,50,51,52,53,54,55]) and successive to a review on IP male cases of 2012 [30]. We included the data concerning the description of anomalies of 39 IP male patients belonging to our collection [56]. All patients showed at least two of the four classical post-natal sequential cutaneous linear eruptions on the skin, which are the major criteria for diagnosis of IP. Overall, the data reported indicate that IP male patients presented with odontologic (14% of male IP with partial anodontia, and conical teeth), ophthalmologic (29% of IP male with retinal detachment, retinopathy, optic nerve atrophy) symptoms and, more importantly, they presented with severe CNS affections such as epilepsy, psychomotor delay, spastic paralysis, brain atrophy, and microcephaly, as reported in 26% of cases. Nevertheless, IP condition in male is rarer then in female, currently a total of 332 males have been reported in literature with a frequency of symptoms of 28% CNS, 39% dental-oral, and 22% eyes defects (Table 1). The total of female with IP in the 1906–2020 period was 2930 females (90% females, 10% males).

## 4. Pitfall in Demonstrating Mosaicism in IP Males

The recent identification of mosaicism for *NEMO* mutation in sperm cells from IP patients has revealed a risk of recurrence of the disease. The challenges of identifying such rare mosaic mutations depends on the tissue distribution of the mutation. Normally, mutations that occur earlier in the development will affect multiple tissues, including germline cells, and can be easily identified. However, detection of *NEMO* mutation in IP mosaic males presents several criticisms.

The first potential pitfall in identification of a mosaicism in male with IP is to test the sample tissue containing cells harboring the *NEMO* mutations as the diagnostics conducted on peripheral blood cells could be ineffective because of multiple rounds of self-renewal during hematopoiesis. The genetic analysis based on the quantitative evaluation of the mutant *NEMO* allele in the DNA from different tissues available has revealed mosaicisms in freshly picked up skin lesions, urine, and sperm [25,26,42]. 

**Figure 2:**
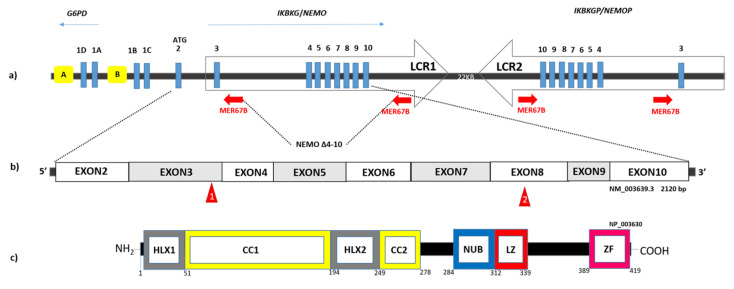
The *NEMO/IKBKG* locus. (**a**) Schematic representation of the genomic organization of the *NEMO/IKBKG* gene and pseudogene (*IKBKGP*) in Xq28 region and the pathogenic *IKBKGdel* (*NEMO Δ4-10*) deletion produced by recombination between MER67B repeats (red arrows). The gene is part of a 35.7 kb segmental duplication (Kilobase) containing two LCRs (Low Copy Repeats) regions shown as two opposite arrows: 1 and 2 (LCR1 chrX: 153784097-153819590; LCR2 chX: 153841350-153877149, UCSC 2013). The *IKBKG* gene is composed of nine coding exons (blue rectangles) and four non-coding alternative exons (1D, 1A, 1B, 1C). Transcription is directed by the bidirectional promoter B or by the unidirectional promoter A (yellow boxes), located in intron 2 of the *G6PD* gene. (**b**) Schematic view of the transcription of *IKBKG* gene and of the small mutations found in the IP male are shown in the red triangles (p.Gln132* and p.Gln313*) [26,42]. (**c**) Schematic view of the NEMO/IKKγ protein and its structural domains from the N-terminal to the C-terminal: HLX1, *Helical domain* (aa: 1-51); CC1, *Coiled Coil* (aa: 52-194); HLX2, *Helical domain* (aa: 195-249); CC2, *Coiled Coil* (aa: 250-278); NUB, *NEMO Ubiquitin Binding* (aa: 284-312); LZ, *Leucin Zipper* (aa: 312-339); ZF, *Zinc Finger* (aa: 389-419).

The second pitfall is due to methods of detection. Sensitive detection and quantification of the Copy Number Variation (CNV) in IP locus (the recurrent deletion of *NEMO* exon 4–10) by using QPCR is the only effective method in the diagnosis. The only three Loss-of-function mutations of *NEMO* found in IP males are: the recurrent exon 4–10 deletion, p.Gln132* and p.Gln313* [26,41]. Powerful genomic tool such as NGS was unproductive [57,58] (Figure 2).

A third potential pitfall is linked to the use of primary cells from skin biopsy. A failure in demonstrating mosaicism through the use of cultured keratinocytes or fibroblasts from the lesioned skin of a boy with IP has been reported. This is due to the likelihood that cells harboring or activating the mutant *NEMO* gene have a selective survival disadvantage. As a result, in these cultures only the wild type *NEMO* allele is detectable even if both normal and abnormal keratinocytes are captured in the original biopsy section [26].

Overall, IP diagnosis in males requires the analysis of fresh tissue before the counter-selection of the *NEMO* mutant cells takes place. This last evidence excludes the possibility to use patient derived cells for modelling the disease in vitro. Recently, the higher levels of cells harboring *NEMO* mutations found in the sperm (up to 35% of cells) of mosaic males explain the recurrence of the disease in the affected siblings born with an identical variant [26,27]. 

The combined somatic and gonadal mosaicism in IP males suggests a non-clonality of the germ line in which the mosaicism arose at a totipotent cell stage of development within the first few cell divisions of the embryo by postzygotic mutation (Figure 1B). Thus, the timing of the mutation should affect the abundance of the mutant cells, the presence of the mutation in the germ cells, and, by extension, the potential recurrence risk for the same mutation to be transmitted to multiple offspring.

## 5. NEMO Deficiency and Cell Death: Implications for the IP-Phenotype 

NEMO is the regulatory subunit of the IKK complex that also comprises the catalytic subunits IKK-1 and -2, the kinase complex essential for the canonical NF-κB activation. The family of NF-κB transcription factor exerts pro-survival and pro-inflammatory activities and its transcriptional program is specifically adapted depending on tissue contexts. 

**Figure 3:**
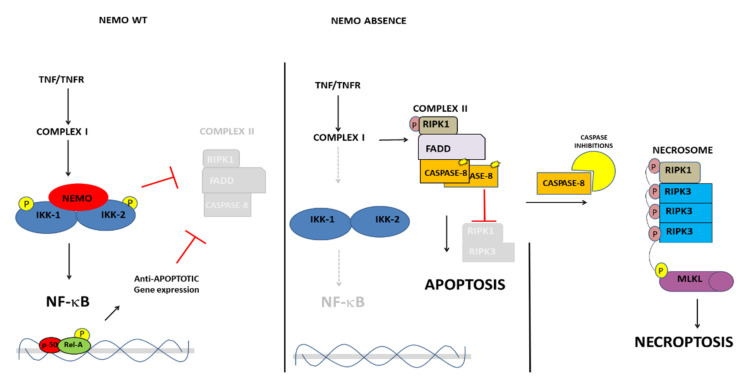
The model of anti-apoptotic NEMO activity and the different complexes formed upon TNFR engagement. In the presence of NEMO-WT (left panel) or in the absence of NEMO, apoptosis (central panel) and necroptosis (right panel) induction.

The effect of NEMO deficiency has been well characterized in the context of inflammatory cytokines triggering, more in detail, the tumor necrosis factor (TNF) receptor-1 (TNFR1) signaling. In physiologic condition, TNF-R triggering induces recruitment and full activation of IKK at the level of the membrane in a multiprotein complex called TNF-R Complex I (Figure 3). The subsequent phosphorylation of the inhibitor of NF-κB (IκB) proteins by IKK allows NF-κB proteins to induce upregulation of several genes mainly implicated in the inhibition of the cytoplasmatic TNFR-complexes, inducing apoptosis via FADD-Caspase-8, or necroptosis by the RIPK1-RIPK3-MKLK axis. Studies on human and mouse embryonic fibroblasts harbouring *NEMO* deletion demonstrated a high sensitivity to cell death [9,59,60]. 

In male mice, *NEMO* ablation caused lethality during embryo development due to cell death in multiple tissues, while heterozygous females manifested a skin IP-like phenotype [59,61,62,63]. The embryonic lethality caused by *NEMO* deficiency in males seems to be determined, at least in part, by RIPK1 kinase activity-dependent TNF-induced death of cells [64]. During embryo development, IKK and NF-kappaB signaling act at multiple levels in the inhibition of cell death both by directly modulating upstream components of the cell death machinery such as Receptor Interacting Protein Kinase-1 (RIPK1), but also by inducing pro-survival gene expression [65]. 

An inflammatory response requiring TNFR1 has been shown to be essential also for the pathogenesis of the skin lesions in IP female mice, suggesting that TNF-mediated inflammation is an obligatory component of the disease [59,66]. Noteworthy, chimeras generated from NEMO knockout (KO) ES cells develop IP-like skin lesions but chimeric mice do not possess any ES cell-NEMO-Ko derived lymphocytes [63]. The latter condition is very similar to the one observed in mosaic human male where a counterselection against NEMO-deficient cells is necessary for sustaining and complete embryo development in some tissues such as in the fetal liver. Whether or not an equivalent mechanism operates also in other IP-affected tissues (CNS or retina) is an open question. Ultimately, a greater appreciation of the effects of cell death in a specific context will improve our understanding of the altered processes, which are on the basis of symptoms of the most severe IP cases, with global developmental delay, seizures, and blindness.

## 6. Final Remarks on the Genetics of IP

“Defined by human geneticists according to its role in the X-linked disorder incontinentia pigmenti, *NEMO* is an X-linked gene with a number of fascinating features that make it a marvelous example for teaching” this definition [67] describes at the best the manifold characteristic of NEMO. Indeed, the local architecture of the IP/NEMO locus, embedded in a region of low and high copy repeats with a very close pseudogene copy and an overlapping gene, G6PD, sharing the bidirectional CpG promoter (Figure 2A) has predisposed it to many different mutations and aberrant recombinations occurring both at the meiotic and mitotic/somatic division, which underline the sporadic cases and mosaicisms.

## 7. Conclusions

In conclusion, mosaic mutations underlie IP disease, where three different forms of mosaicisms might coexist: (a) functional X linked mosaicism; (b) germline mosaicism (also known as gonadal mosaicism); and (c) somatic mosaicism. 

Quite surprising, gonadal mosaicism due to somatic NEMO mutation in males with IP arise in the embryo and persist until adulthood diversely from other tissue (i.e., blood) where the levels of mosaicism are undetectable in adult (Figure 1A). Moreover, a different mechanism of cell death apoptosis and/or necroptosis could operate the cell selection, inducing inflammation and clarence at least in the skin. Distinguishing between these two mechanisms is noteworthy for disease therapy, as the type of cell death specifies whether or how the immune system is engaged by dead cells. 

## Figures and Tables

**Table 1:** The main findings of IP male patients and recurrent anomalies (2013–2020).

	* IP Patients	** Ocular Disease	Dental Anomalies	*** Neurological Changes
**Total**	91 *	26	13	24
**Number of IP patients** **undergoing molecular investigation**	77	23	11	21
**Positive Genetic test**	32	13	6	12
**Negative Genetic test**	45	10	5	9

* Two male IP patients also were Klineferter. ** Ocular anomalies are thus distributed: 12 cases of retinal detachment/blindness, 4 cases of vision changes, 6 cases of retinal vascular disease, 2 cases of optic nerve atrophy, 2 cases of pigmentation changes, 2 cases of foveal hypoplasia, and 1 case of blue sclera. *** CNS anomalies are thus distributed: 14 cases of MRI anomalies, 4 cases of convulsions, 9 cases of epilepsy, 7 cases of psychomotor delays, 2 cases of hypertonia, 1 case of hypotonia, 1 case of intracranial hemorrhages (ICHs), and 3 cases of contact disorder.
